# The International Society of Surgery

**Published:** 1907-03-02

**Authors:** 


### The Hospital

A Journal of

The Medical Sciences and Hospital Administration.

Vol. XLI.—No. 1068. SATURDAY, MARCH 2, 1907.

### THE INTERNATIONAL SOCIETY OF SURGERY.

The International Society of Surgery holds an interesting place amongst the societies of the world, and if it be well managed it appears that it will be useful and successful. It was established in Brussels in 1902, with a limited number of members. It meets once in three years, the last meeting being in 1905, and the next in 1908. His Majesty Leopold II., King of the Belgians, is patron, and His Excellency Professor Czerny of Heidelberg is President. The Society is governed by a permanent International Committee composed of a delegate from each country, the ex-Presidents of the Congress, a Secretary, and a Treasurer. Every country is represented by three members, of whom the delegate is one, and these three members form a National Committee charged with the selection of candidates for membership and the conduct of the local business of the Society in the intervals of its meetings. The subjects considered at the triennial meetings of the Society are thought out carefully beforehand; members are invited to take a part in them who are specially qualified to do so, and their contributions are printed and circulated some weeks before the actual meeting. Every member of the Society therefore is fully cognisant of the views which will be brought forward, and with the printed paper of the speaker in his hand he is able to endorse or differ from the views advanced. The subjects considered at the meeting in 1905 were: "The Value of the Examination of the Blood in Surgery," "The Treatment of Prostatic Hypertrophy," "Surgical Interference in Non-malignant Disease of the Stomach," "The Treatment of Tuberculous Arthritis," and "The Treatment of Peritonitis"—all good practical subjects, which elicited excellent discussions in English, French, and German.

The meeting of the Society in September, 1908, promises to be equally fruitful. It has been decided to organise an exhibition illustrative of cancer and its treatment. With a view to secure a systematised method of procedure in the construction of reports relating to specimens, some suggestions have been prepared by Dr. Jules Dollinger, Professor of Clinical Surgery in the Royal University of Buda-Pesth, who is President of the International Museum of Cancer. Specimens for exhibition will be received by Professor Depage in Brussels, and the exhibition is not necessarily confined to members of the Society. The Congress will be so arranged as to allow exhibitors to make such demonstrations as may appear desirable by the Society during its meeting. No attempt has hitherto been made to hold an international museum of cancer, and the idea is a good one, though it remains to be proved whether it can be carried out on any large scale. Such a museum might be made to show, for instance, the real existence of contact carcinomata, which are often doubted because they are rarely seen; the history and course of endotheliomata, because as yet surgeons know very little about them; whilst the new growths of synovial membranes are urgently in need both of study and of illustration. The Congress has already decided the headings under which the cancer question is to be considered. In brief, they are: The results of treatment by modern operative methods; fresh methods of treatment; the result of treatment by methods other than operative, such as light treatment; serum therapy, and the like. It is further considered desirable to ascertain how far the public ought to be instructed in the early signs of cancer with a view to earlier diagnosis, and consequently of earlier operation. Apart from cancer, the other subjects for consideration will be the surgery of the liver, of the spinal cord, and of herniæ; and there will be a discussion on anæsthesia.

It is very clear from this programme that the cancer discussion will be the most full, and the last question will be by no means the least interesting. Early recognition in cases of cancer means early removal in the majority of cases, with corresponding increase in the prospect of life. But too often recognition is delayed because the patient does not apply for a diagnosis until his attention is drawn to some unmistakable sign, and this is due to ignorance rather than to carelessness. There is a prevailing opinion that cancer is always painful from the beginning, whereas it is really painless in the great majority of cases; most people are unaware of the precancerous conditions in which the continuance of irritation is extremely likely to end in the formation of a malignant growth. Were the public properly instructed in these matters, many tongues would be either saved or removed with a better chance of success. Many persons with cancer of the large intestine would submit to a local removal rather than wait until a colotomy was alone possible; and many a woman with cancer of the breast would not need to be told that she had postponed too long her visit to the surgeon because she had not suffered pain. Such information could be disseminated readily enough by the exercise of a little tact, without in any way creating a panic, and it would be very beneficial to all classes of the community.

